# Strength in Numbers: an international consensus conference to develop a novel approach to care delivery for young adults with type 1 diabetes, the *D1 Now* Study

**DOI:** 10.1186/s40900-017-0076-9

**Published:** 2017-12-04

**Authors:** M. C. O’Hara, L. Hynes, M. O’Donnell, C. Keighron, G. Allen, A. Caulfield, C. Duffy, M. Long, M. Mallon, M. Mullins, G. Tonra, M. Byrne, S. F. Dinneen, with the D1 Now Type 1 Diabetes Young Adult Study Group

**Affiliations:** 1 School of Medicine, National University of Ireland, Galway, Galway, Ireland; 2 Health and Wellbeing Division, Health Service Executive, Merlin Park University Hospital, 2nd Floor, Block A H91 N973, Galway, Ireland; 3 SPLAT (Pediatric Lab for Adherence and Transition), West Virginia University, Morgantown, USA; 4 Member of the D1 Now Young Adult Panel, Galway, Ireland; 5 School of Psychology, National University of Ireland, Galway, Galway, Ireland; 6 Centre for Diabetes, Endocrinology and Metabolism, Galway University Hospitals, Galway, Ireland

## Abstract

**Plain English summary:**

Many young adults with type 1 diabetes struggle with the day-to-day management of their condition. They often find it difficult to find the time to attend their clinic appointments and to meet with their diabetes healthcare team. Young adults living with type 1 diabetes are not routinely involved in research that may help improve health services other than being invited to take part in studies as research participants. A 3-day international conference was held in Galway in June 2016 called “Strength In Numbers: Teaming up to improve the health of young adults with type 1 diabetes”. It aimed to bring together people from a broad variety of backgrounds with an interest in young adults with type 1 diabetes. Young people with type 1 diabetes came together with healthcare professionals, researchers, software developers and policy makers to come up with and agree on a new approach for engaging young adults with type 1 diabetes with their health services and to improve how they manage their diabetes.

The people involved in the conference aimed to reach agreement (consensus) on a fixed set of outcome measures called a core outcome set (COS) that the group would recommend future studies involving young adults with type 1 diabetes to use, to suggest a new approach (intervention) for providing health services to young adults with type 1 diabetes, and to come up with health technology ideas that could help deliver the new intervention. Over the 3 days, this diverse international group of people that included young adults living with type 1 diabetes, agreed on a COS, 3 key parts of a new intervention and 1 possible health technology idea that could help with how the overall intervention could be delivered.

Involving young adults living with type 1 diabetes in a 3-day conference along with other key groups is an effective method for coming up with a new approach to improve health services for young adults with type 1 diabetes and better support their self-management.

**Abstract:**

**Background**

A 3-day international consensus meeting was hosted by the *D1 Now* study team in Galway on June 22–24, 2016 called “Strength In Numbers: Teaming up to improve the health of young adults with type 1 diabetes”. The aim of the meeting was to bring together young adults with type 1 diabetes, healthcare providers, policy makers and researchers to reach a consensus on strategies to improve engagement, self-management and ultimately outcomes for young adults living with type 1 diabetes.

**Methods**

This diverse stakeholder group participated in the meeting to reach consensus on (i) a core outcome set (COS) to be used in future intervention studies involving young adults with type 1 diabetes, (ii) new strategies for delivering health services to young adults and (iii) potential digital health solutions that could be incorporated into a future intervention.

**Results**

A COS of 8 outcomes and 3 key intervention components that aim to improve engagement between young adults with type 1 diabetes and service providers were identified. A digital health solution that could potentially compliment the intervention components was proposed.

**Conclusion**

The outputs from the 3-day consensus conference, that held patient and public involvement at its core, will help the research team further develop and test the *D1 Now* intervention for young adults with type 1 diabetes in a pilot and feasibility study and ultimately in a definitive trial. The conference represents a good example of knowledge exchange among different stakeholders for health research and service improvement.

## Background

The global incidence of type 1 diabetes is increasing. Although much of the focus has been on childhood cohorts, a significant annual increase has also been reported for young adults [1]. A recent international comparison study of glycaemic control indicated that young adults living with type 1 diabetes frequently experience poor outcomes with 15–24 year olds most likely to have HbA_1c_ values greater than 58 mmol/mol (> 7.5%) [2].

In recent years it has been acknowledged that young adults with type 1 diabetes should be recognised as having different needs and facing different challenges, compared to younger and older cohorts living with the condition. Young adulthood was highlighted as an area requiring specific research in a position statement from the American Diabetes Association (ADA) on Transition from Paediatric to Adult Care [3].

Disengagement from diabetes services is common among young adults with type 1 diabetes and has been associated with increased risk in this population [4, 5]. In a recent systematic review of barriers and facilitators to clinic attendance among young adults with type 1 diabetes, continuity of care and positive transition experiences (from paediatric to adult services) were identified as facilitators of clinic attendance [6]. Researchers in Galway, Ireland, developed a theory of clinic attendance based on a series of in-depth interviews with young adults and service providers [7]. It highlighted that regular clinic attendance behaviour occurred primarily as a result of forming good relationships between young adults and healthcare professionals. These relationships with members of the diabetes team appeared to be formed through interactions such as attendance at a structured education programme or in-patient stays, and made it more likely young adults would seek support from the diabetes team and attend appointments.

The study group based across the National University of Ireland, Galway (NUI Galway) and Galway University Hospitals campuses began a research study in 2014, funded by the Health Research Board, in response to the problems highlighted by an audit of young adults (18–25 years old) attending their service (and international evidence). In brief, the local audit reported poor glycaemic control, poor clinic attendance, frequent Emergency Department attendance in some young adults and one death [8]. A patient and public involvement (PPI) framework guided this research meaning the research would be carried out ‘with’ or ‘by’ young adults with type 1 diabetes rather than ‘to’, ‘about’ or ‘for’ them [9]. Using the Medical Research Council guidance for developing and evaluating complex interventions [10] and the Behaviour Change Wheel for characterising and designing behaviour change interventions [11], the study aimed to establish an evidence base for developing a new intervention for young adults living with diabetes, called *D1 Now*, under five key work streams as outlined below.

First, a PPI panel, known as the Young Adult Panel (YAP), consisting of 8 young adult service-users aged between 18 and 25 years old with type 1 diabetes was formed using a successful model of involving users in service design developed by Jigsaw, Galway [12–14]. Following an open consultation evening held in Jigsaw Galway, (a community-based youth mental health service that is committed to youth engagement), the YAP was recruited to work as co-researchers with the study team [15]. The PPI element of the study resulted in meaningful involvement and teamwork between young adults, researchers and service providers. The YAP has made significant contributions to all aspects of the development study, including designing and reviewing study materials and disseminating results. The YAP members developed the topic guides for the qualitative work stream of this study, and the participant consent and information forms. The YAP members also participated in disseminating the study’s findings by submitting scientific abstracts to national conferences, being invited speakers at 2 national conferences, being interviewed for local newspapers and radio and by entering national science competitions. Two YAP members were elected to sit on the study’s steering committee and 2 on the organising committee for the consensus conference described in this report.

Second, a systematic review of all interventions aimed at improving clinical, behavioural and psycho-social outcomes for young adults with type 1 diabetes was conducted [16]. Continuity, support, education and tailoring of interventions to young adults were the most common themes across the studies reviewed. Due to the heterogeneity between the studies, it was not possible to derive a combined effect size or to make conclusions regarding the efficacy of existing interventions for improving outcomes in this population.

Third, Discrete Choice Experiment (DCE) methodology was utilised to explore the preferences of young adults with type 1 diabetes and service providers related to the delivery of outpatient diabetes clinics and the provision of support between clinic appointments. DCE is a technique commonly used in health economics to elicit patient preferences for healthcare services [17]. It is an attribute-based measure of benefit; attributes could include waiting times, health service providers available at a particular clinic, or when appointments are available (for example weekday or weekend days). The DCE model is based on the assumption that healthcare services can be described by their attributes and an individual’s valuation depends upon the levels of these attributes [17]. The DCE methodology has previously been used to assess preferences for diabetes management among people with type 2 diabetes [18].

Fourth, a qualitative engagement study was also conducted with key stakeholders to understand the factors that influence diabetes self-management and how services and support could be improved (this manuscript is currently being prepared for publication by co-author LH). Focus groups were conducted with young adults with type 1 diabetes, and interviews were conducted with parents of young adults with type 1 diabetes and healthcare providers, in Galway, Dublin and Belfast.

Finally, the output from the development work completed in this study was synthesised by the research team to form the evidence base to guide a consensus process conducted over the course of a 3-day conference. Consensus activities included a conference and an expert panel meeting involving representative stakeholder groups. Delegates, including young adults with type 1 diabetes, researchers and diabetes service providers, were facilitated to agree on the components of a complex behaviour change intervention to improve self-management among young adults living with type 1 diabetes.

The present article describes the process and outcomes of this stakeholder-led consensus conference and process called “Strength In Numbers: Teaming up to improve the health of young adults with type 1 diabetes”. By conducting a stakeholder-led consensus process it was hoped that an intervention would be developed that was acceptable to young adults and service providers, increasing the likelihood of engaging stakeholders in the evaluation and implementation of the proposed intervention.

## Methods

A consensus process, including a conference and series of stakeholder meetings were conducted to present and discuss the evidence gathered in the *D1 Now* intervention development study described above. The aim of the process was to reach agreement on components of a complex intervention to improve self-management among young adults with type 1 diabetes. A consensus process was the approach chosen in line with the PPI framework of this study.

### Participants

Delegates and conference speakers were identified and invited to represent the relevant stakeholder groups. Conference registration was free of charge and was advertised widely through a social media campaign using print media, email alerts, Facebook and Twitter. Relevant national and European organisations also publicised the event, such as Diabetes Ireland, Irish Endocrine Society, European Association for the Study of Diabetes, Irish Nutrition and Dietetic Institute, Health Research Board and the Irish Diabetes Nurse Specialist Association. Table 1 details the number of participants across the various stakeholder categories that attended over the 3 days of the conference.

**Table 1:** Conference participants by stakeholder category

Participant categories	COS (June 22)	Conference Speakers & Chairs (June 23)	Conference Attendees (June 23)	Expert Panel (June 24)	Hackathon (June 24)
Young adults with type 1 diabetes	3	3	16	4	6
Health/Research Psychology	3	4	8	3	4
Engineer/Software Developer	0	1	11	0	3
Digital Health/Health Technology	0	1	8	0	8 (5 plus 3 facilitators)
Diabetes Nurse Specialists	1	2	9	2	3
Doctors	2	3	9	2	0
Dietitians	0	2	6	2	1
Youth Mental Health	0	1	5	0	0
Policy Makers	1	0	5	3	1
Researchers	2	0	13	2	0
Not specified (delegates did not answer this section of the registration form)	0	0	20	0	0
Total	12	17	110	18	26

### Consensus conference

The 3-day international consensus conference called “Strength In Numbers: Teaming up to improve the health of young adults with type 1 diabetes” had four principle components:

- First, on the day proceeding the conference a core outcome set (COS) was compiled based on data collected from two online surveys and discussion with expert stakeholders to reach a consensus on a core set of measures that could be used in future intervention studies
- Second, keynote speakers from 4 key areas gave presentations covering innovations to service delivery, self-management support, harnessing the power of social media and digital technology and engaging young adults in health service research. Themes emerging were flexibility of services, tailoring of interventions for young adults and the importance of listening and communication.
- Third an expert panel discussed 3 focus areas with potential to improve self-management; (i) the way young adults are introduced to the adult diabetes clinic, (ii) attendance at diabetes clinic appointments and contact between appointments and (iii) building relationships between young adults and healthcare providers.
- Finally, a Hackathon took place, where computer programmers along with software developers met key stakeholders (young adults with type 1 diabetes, healthcare professionals and researchers) to form teams which collaborated on technology solutions to better support young adults with type 1 diabetes [19, 20]. Four novel ideas were pitched to the expert panel, that chose a winner. The winner was an idea called “SnapD1”, this would use a channel on the popular social media app “Snapchat” and it would feature motivational, factual and useful content daily.

## Results

### Core outcome set (COS) – June 22, 2016

The COS process was funded by the Irish Research Council, it involved a two-phase online Delphi survey administered ahead of the conference [21, 22]. A list of all measures used in published interventions involving young adults with type 1 diabetes were compiled (*n* = 87) and grouped in 7 domains (lifestyle, quality of life, diabetes clinics, medical, blood glucose, treatment preferences in relation to diabetes and intervention-related outcomes) by a researcher in the School of Psychology, NUI Galway. Young adults living with type 1 diabetes, healthcare professionals and researchers in the area were targeted to complete an online survey. Stakeholders completed the online survey over two rounds (*n* = 127 in survey 1, 34 were young adults with type 1 diabetes and *n* = 81 in survey 2, 17 were young adults with type 1 diabetes), to rate and re-rate the importance of each of the outcomes on a scale of 1–9, where 9 was the most important. Each outcome was categorised into one of three groups based on its overall rating. Outcomes rated by 70% of participants as 8+ were grouped into Category A. Outcomes rated by 70% of participants or more, as 6 or less were grouped in Category C and excluded from further discussion. All other outcomes (rated 8) were grouped into Category B, these warranted further discussion. On June 22, expert stakeholders (*n* = 12, see Table 1 for a breakdown) re-rated for a third time the outcomes which fell into Category A and Category B to reach consensus on a core set of measures.

A COS is an example of a multi-perspective stakeholder engagement process. During this consensus event 8 core outcomes were agreed. This process and its outcomes have been described in greater detail in a manuscript which has been submitted for publication separately.

### Strength in Numbers conference – June 23, 2016

Stakeholders (*n* = 110) in young adult diabetes management from across the island of Ireland and from Canada, England, Scotland, Japan, Denmark and Australia were invited to attend this international conference with an emphasis on listening to the voice of young adults living with type 1 diabetes.

Over 14% of the conference delegates were living with type 1 diabetes. Keynote speakers focused on four key areas; (i) innovations in service delivery and re-design, (ii) self-management support, (iii) harnessing the power of digital technology and social media and (iv) engaging young adults in health services research. These key areas were chosen to facilitate knowledge exchange and discussion that was deemed most relevant to the overall intervention development process, based on the development study findings. Out of the 11 speakers invited to present at the conference, two were young adults living with type 1 diabetes. The prominent themes that emerged from the presentations were flexibility, tailoring services to the needs of young adults and the value of services designed based on consultation with young adults. The complexity of living with type 1 diabetes and approaches needed to address the unique needs of young adults were emphasised. Listening and communicating were cited on numerous occasions, both during presentations and audience discussions, as core activities when working with young adults to understand where they are coming from and what diabetes services can do to support them.

### Strength in Numbers expert panel – June 24, 2016

The development study resulted in the identification of three focus areas with the potential to improve self-management support for young adults with type 1 diabetes. Prior to the consensus conference, expert panel delegates (see Table 1 for a breakdown of delegates) were sent information summarising the development study, the focus areas for improving self-management support, and an outline of their role on the expert panel. The focus areas to guide the consensus process were:

1. The way young adults are introduced to the adult diabetes clinic.
2. Attendance at diabetes clinic appointments, the booking system and contact between appointments.
3. Building relationships between young adults and their service providers.

Expert Panel members were split into 3 representative teams (with 1 or 2 young adults with type 1 diabetes on each team). In session 1 each team was asked to examine and debate 2 of the 3 focus areas listed above, over two rounds of discussion, first within their individual team and then with the whole group. Guidance on intervention development using the Behaviour Change Wheel recommends that a structured behavioural analysis is first conducted, to understand the determinants of a target behaviour [11]. Using this structured behavioural analysis, each group identified up to 3 specific strategies which could be used to address their assigned focus area. Each focus area had 6 strategies in total discussed. Following the identification and discussion of possible strategies, each one was assessed by the groups, using a rating of high, medium or low, according to the criteria of impact on young adult self-management, how feasible the strategy was and the potential for each strategy to have positive knock-on effects (positive knock-on effects could include increased clinic efficiency if an online booking system was implemented to improve young adult’s clinic attendance rates). The 2 strategies with the highest ratings per focus area were then discussed in more detail.

The strategies deemed to be most promising for addressing each focus area were:

1. **The way young adults are introduced to the adult diabetes clinic**

- Recruit a ‘youth worker’ to act as guide and advocate for young adults.
- Launch a website to orientate young adults to the adult service and to facilitate contact with staff as required.

2. **Attendance at diabetes clinic appointments, the booking system and contact between appointments**

- Create an online flexible appointment booking system and pre-consultation agenda-setting tool to support/ facilitate engagement among young adults.
- Diabetes service providers will communicate with young adults to discuss and agree the purpose of clinic appointments. This will be carried out during an initial consultation and reviewed online and face-to-face, as required.

3. **Building relationships between young adults and service providers**

- Create an agenda-setting tool to be used before and during consultations to facilitate relationship development and collaborative diabetes management.
- Recruit a youth worker to proactively reach out to young adults using a holistic approach to addressing their needs.

The following important themes emerged during discussion; (i) choice of when to transition from paediatric to adult services, (ii) employing strategies used in social psychology and marketing to engage young adults, (iii) flexibility of and accessibility to services, (iv) collaborative approach to diabetes management, (v) continuity of care, (vi) the needs of young adults should be frequently reviewed and (vii) multiple modes of contact and engagement including face-to-face and online should be utilised.

In session 2 Expert Panel teams discussed plans and barriers for implementing their most promising strategy before feeding back to the group.

The most promising strategies for each focus area addressed service organisation (e.g., introducing a flexible online booking system, employing a youth worker), service providers (e.g., adapting a young-adult-centred collaborative approach to care delivery, delivering services in ways other such as online) and young adults (engagement and activation with diabetes services).

The barriers to implementing the identified strategies included: funding, acceptance and engagement and training of service providers and young adults. Logistical barriers were also identified such as planning, time, staffing levels and access to technology within the Irish national health service, and identification and accountability of appropriate youth workers. Solutions for overcoming barriers included engaging with young adults themselves, learning from other service-user groups seeking similar changes in other chronic condition areas, using marketing and social psychology approaches to enhance engagement, pilot testing strategies, collecting feedback, building flexibility within strategies and addressing technology gaps.

### 'Strength in Numbers' Hackathon – June 24, 2016

A Hackathon is a dynamic approach to creating solutions to well defined problems in a short period of time, involving collaboration by relevant stakeholders such as computer programmers and business people [19]. In this case stakeholders were brought together, including computer programmers, young adults with type 1 diabetes, researchers and diabetes service providers, to identify technology solutions to fit within an intervention to improve outcomes for young adults. The Health Hackathon Handbook [20] informed the planning and implementation of the Strength in Numbers Hackathon.

Hackathon participants were issued with a “problem statement” two weeks ahead of the event, that reflected the information shared with the expert panel members. A problem statement is a concise description of the problem and needs that are to be addressed by a problem solving team, and is the starting point of a Hackathon, to ensure that the diverse participants are facilitated to understand the Hackathon target and bring their personal strengths to the brainstorming activity.

Self forming teams were established from the mixed stakeholder group, around 5 proposals deemed by the group to be most promising, during the pre-Hackathon meeting held on the evening of June 23. Six of the 26 people who participated in the Hackathon were young adults living with type 1 diabetes. The following day teams worked collaboratively on their proposals, led by expert facilitators from the HSE Office of the Chief Information Officer and the NDRC Catalyser. Both agencies specialise in supporting health service innovation and regularly facilitate Hackathons. Participants came from organisations/ groups such as the *D1 Now* YAP, Diabetes Ireland Ambassadors (young people with type 1 diabetes who work with the national diabetes charity in Ireland) Hewlett Packard Enterprise, Blackstone LaunchPad and patientMpower, as well as staff from 6 hospitals across Ireland and 3 universities in Ireland and the UK.

The Hackathon was an energetic and creative meeting and resulted in four interesting and novel ideas [23]. Each Hackathon team pitched their idea to the Expert Panel, who then chose the winner of the “Best Pitch Award”. Judging criteria were based on 2 main elements; (i) suitability for a young adult population and (ii) enhancing relationships between young adults and their healthcare providers. Solutions pitched included developing a transition app to assist smoother transition, a diabetes monitoring and communication app and an app to provide a 2-way communication between young adults and service providers in real-time. The winning pitch was called SnapD1, and involved a channel on the popular social media app called Snapchat. SnapD1 sends young adults motivational and informative content that can be personalised and integrates a social network aspect. The creators proposed that SnapD1 could be used to send reminders, facts and top tips as well as providing some young adult generated content and motivational pictures and comments. The research team are working closely with the study’s YAP who are currently working on generating the creative content for SnapD1.

## Discussion

This study demonstrates that it is feasible and valuable to engage stakeholders in a consensus process to develop complex behaviour change interventions. Delivering effective diabetes health services to young adults has proven to be a significant challenge. Suboptimal outcomes associated with this phase of life can lead to negative attitudes on the part of healthcare professional and patients alike in terms of managing diabetes and engaging with services [24]. Traditional clinic-based methods of delivering care have significant limitations and may not be appropriate for supporting self-management and optimal outcomes among young adults.

For example, research has shown young adults often become disengaged from adult diabetes services following transition [5]. Young adults rely on adult diabetes services for diabetes-related and emotional support, due in part to the changing support system associated with young adulthood [25]. Yet, differences in the service they experience after transition and a lack of preparation for these differences, and tailoring of adult services to each young adult, has been reported as a significant barrier to engagement among young adults [26]. Despite the insights held by service providers in relation to the experiences and needs of young adults, the structure of traditional clinics consistently hinder attempts to engage young adults [7].

Research related to innovations in service delivery, particularly patient-centered care delivery illustrates that it is difficult to “re-imagine” care delivery and much easier to continue to deliver care in the same way. The Strength In Numbers conference and consensus process successfully brought stakeholders in young adult type 1 diabetes management together to produce the components of an intervention to improve outcomes in this population. Due to the complexity and challenges that needed to be addressed in defining a new intervention for young adults with type 1 diabetes, a consensus conference of key stakeholders was an ideal forum to adopt.

The events that occurred during our 3-day international conference were intended to help the research team to re-imagine what diabetes health services could look like for young adults. The role of the public and patients is what characterises a consensus conference [27, 28]. Collecting and considering input from stakeholders is recommended in current guidelines for effective intervention development and for research guided by a PPI framework [10, 29]. The Strength in Numbers conference and consensus process resulted in multiple outcomes. First, a strong network of engaged stakeholders was established, that will continue to interact to move towards the collective goal of improving outcomes among young adults with type 1 diabetes. Second, this event produced the components of an intervention protocol that formed the basis of an application for national funding to further develop the *D1 Now* intervention to improve self-management among young adults with type 1 diabetes. The intervention will be modelled and tested in a feasibility study and subsequent randomised pilot before applying for funding to conduct the definitive trial. Third, the conference was the culmination of a PPI study to develop an intervention, which demonstrated the work completed by the research team as well as demonstrating the commitment of the team to PPI.

The conference was successfully organised by a PPI team and greatly enhanced by the level of stakeholder involvement throughout the event. We invited all members of our study’s PPI Young Adult Panel to join the organising committee. Two members joined the organising committee (they were involved in setting the agenda, in developing the delegate packs, and preparing the venue) and another 5 members contributed to the 3 days by volunteering for various roles and responsibilities. This PPI YAP panel consists of 8 young adult (18–25 years old) service-users. They have a combined 108 years experience of living with type 1 diabetes. As well as helping in preparing conference materials, 5 YAP members volunteered to participate during the 3 days by manning the registration desk, chairing a conference session, delivering a keynote presentation and by participating in the COS, Expert Panel and Hackathon. By promoting the event as widely as possible, the study team was able to identify other people living with type 1 diabetes to attend the conference and participate in the Expert Panel and Hackathon.

However, challenges certainly arose in managing and welcoming strong and differing views shared throughout the COS meeting, conference, expert panel meeting and Hackathon. The contributions made to this consensus conference by individuals with type 1 diabetes were a great strength of the event. Discussions arose which challenged all conference delegates, in terms of approaches to research and practice. Future events should carefully consider approaches to truly integrate all stakeholders within dissemination and knowledge exchange events, including appropriate communication and use of terminology to ensure all voices are heard.

Carrying the input from the conference and consensus process through to writing an intervention protocol required a balance between the integration of opinions, consensus, barriers and logistical issues, and existing evidence, that posed a new and considerable challenge to the research team. In managing this balance, new and formal collaborations have been forged between both academics and young adults living with type 1 diabetes. Using scientific frameworks and guidelines to underpin the *D1 Now* intervention development process, our team has successfully been awarded a Definitive Intervention and Feasibility Award from the Health Research Board (Ref: DIFA-2017-034) in Ireland to progress this stakeholder/ PPI-led study.

### Limitations

Approaches to reaching consensus in research have evolved considerably in recent years. The approach taken in the present study was designed to facilitate the planned work, to bring together a wide range of stakeholders, in particularly young adults themselves, and to share knowledge as well as reach consensus. As a result, the approach taken in this study may not be appropriate for all research teams aiming to complete a consensus process as part of intervention development.

As there is a lack of existing research available to guide intervention development aiming to improve outcomes among young adults with type 1 diabetes. Therefore, a relatively exploratory approach, drawing on stakeholder experience, existing evidence and novel areas such as digital health, was chosen to address the aims of this study.

Although considerable efforts were made to invite local, national and international stakeholders involved in the area of young adults and type 1 diabetes, the delegates involved in this consensus conference may not be representative and were likely to be a highly motivated and invested group. Alternative consensus methods such as a Delphi [30, 31] or nominal group [32] approach would not have been appropriate for generating discussion and consensus in relation to specific focus areas of the intervention, that were of interest in the present study.

## Conclusion

This conference allowed young adults with type 1 diabetes and other key stakeholders to discuss relevant topics together, hear each other’s point of view and identify possible solutions to improve how health services deliver care to, and engage with young adults with type 1 diabetes. The consensus process conducted during this conference resulted in the identification of components of an intervention to improve service delivery for young adults with type 1 diabetes. These components were produced in collaboration between stakeholders, increasing the likelihood that this intervention will meet the needs of this population. We believe this approach acts as a template for other clinicians and research teams to work collaboratively with people living with a chronic condition to develop meaningful strategies for service re-design.

## Abbreviations

ADA: American Diabetes Association
COS: Core outcome set
DCE: Discrete Choice Experiment
HSE: Health Service Executive
NUI Galway: National University of Ireland, Galway
PPI: Patient and public involvement
UK: United Kingdom
YAP: Young Adult Panel
